# RNA-seq analysis of lncRNA-controlled developmental gene expression during puberty in goat & rat

**DOI:** 10.1186/s12863-018-0608-9

**Published:** 2018-04-02

**Authors:** Xiaoxiao Gao, Jing Ye, Chen Yang, Lei Luo, Ya Liu, Jianping Ding, Yunhai Zhang, Yinghui Ling, Weiping Huang, Xiaorong Zhang, Kaifa Zhang, Xiumei Li, Jie Zhou, Fugui Fang, Zubing Cao

**Affiliations:** 1 Anhui Provincial Laboratory of Animal Genetic Resources Protection and Breeding, College of Animal Science and Technology, Anhui Agricultural University, 130 Changjiang West Road, Hefei, 230036 Anhui China; 2 Anhui Provincial Laboratory for Local Livestock and Poultry Genetic Resource Conservation and Bio-Breeding, 130 Changjiang West Road, Hefei, 230036 Anhui China; 3 Department of Animal Veterinary Science, College of Animal Science and Technology, Anhui Agricultural University, 130 Changjiang West Road, Hefei, 230036 Anhui China

**Keywords:** lncRNA, Puberty, XLOC_446331, Hypothalamus

## Abstract

**Background:**

Puberty is a pivotal stage in female animal development, and marks the onset of reproductive capability. However, little is known about the function of lncRNAs (long noncoding RNAs) in puberty. Therefore, RNA-seq analysis were performed between goats and rats to clarify the roles of lncRNAs and mRNAs in the onset of puberty.

**Results:**

In the present study, the length of lncRNAs, the length of the open reading frame and the exon count were compared between the two species. Furthermore, functional annotation analysis based on Kyoto Encyclopedia of Genes and Genomes (KEGG) and Gene Ontology (GO) analysis of lncRNAs target genes and differentially expressed mRNA demonstrated the significantly enriched terms, such as AMPK signaling pathway, oxytocin signaling pathway, insulin secretion as well as pheromone receptor activity, and some other signaling pathways which were involved in the regulation of female puberty. Moreover, our results of siRNA interference in vitro showed the candidate lncRNA *XLOC_446331* may play a crucial role in regulating female puberty.

**Conclusion:**

In conclusion, the RNA-seq analysis between goat and rat provide novel candidate regulators for genetic and molecular studies on female puberty.

**Electronic supplementary material:**

The online version of this article (10.1186/s12863-018-0608-9) contains supplementary material, which is available to authorized users.

## Background

Puberty is a transitional stage for an animal from the immature sexual state to the mature sexual state, and it marks the onset of reproductive capability [1]. Puberty is well known to be controlled by a range of complex factors and their interactions, including genetic, metabolic, neuroendocrine, nutritional, and environmental factors [2]. It is also well known that the hypothalamic-pituitary-gonadal (HPG) axis plays an important role in the regulation of genes such as *Kiss-1/GPR54*, *TAC3*, and *TACR3* [3, 4], *MKRN3* [5]. Moreover, several studies have revealed that a positive energy balance was required for GnRH (gonadotropin releasing hormone) activation at puberty, such as the insulin signal. As previously reported, insulin signaling proteins are widely detected throughout the hypothalamus, and insulin signaling has been suggested to participate in the regulation of reproduction [6, 7]. Otherwise, blockade of insulin signaling in the brain led to delaying in the puberty of mice [8]. Moreover, significant expression levels of IGFBP5 in mammary tissue suggested that IGFBP-5 may be critical for postnatal mammary development [9]. Decreasing the age at puberty could reduce the cost of developing replacement nanny goats. Previous research has reported hundreds of differentially expressed genes in hypothalamus of pubertal Liaoning cashmere (LC) and Jining grey (JG) goats, and these genes were also involved in both neuroendocrine and energy metabolism [10]. Recently, epigenetic mechanisms of transcriptional regulation have been found to play a crucial role in the onset of puberty in female rats [11]. LncRNAs are the key players in epigenetics, and have been shown to participate in reproduction. Studies thus far have primarily identified lncRNAs in humans and mice and investigated them in detail [12, 13]. Recent studies on mammalian lncRNA datasets include bovine [14–16] and porcine [17, 18] lncRNAs in muscle and skin. The lncRNA HongrES2 was involved in normal sperm maturation in rat epididymis [19]. Another study screened numerous lncRNAs that participated in preimplantation development in mice embryos [20]. The determination of puberty onset and evaluation of reproductive status could be performed by concentrations of Cameroon Dwarf goats serum IGF-1 during prepubertal and pubertal periods [21]. In addition, previous research among ncRNAs and mRNA in ovarian transcriptomic study clarified that lncRNAs were involved in sheep fecundity [22]. We also have screened out some differentially expressed lncRNA in pubertal goat hypothalamus [23]. Therefore, we inferred that lncRNAs played prominent roles in the onset of puberty. In the present study, we carried out simultaneous RNA-seq analysis of goats and rats to explore the lncRNAs participating in the onset of puberty. The findings of this study may contribute to further research on puberty.

## Methods

### Preparation of samples

Adult Sprague Dawley rats were purchased from the Experimental Animal Center of Anhui Medical University and allocated into breeding pairs. Sprague Dawley rats were housed under standard conditions (12:12 h light-dark cycle with lights on between 06:00 and 18:00 h; temperature, 22 ± 1 °C; rat food and water provided ad libitum). The onset of vaginal opening in rats was considered the mark of puberty [24]. The pubertal rats (*n* = 3) on 35 d postnatal [25] and prepubertal rats (*n* = 3) on 25 d postnatal were decapitated in the healthy physiological stage after deep anesthesia with pentobarbital sodium. Hypothalamic tissues of rats were surgically removed in the same estrus cycle stage (Additional file 1), and frozen in liquid nitrogen immediately. The samples were kept at − 80 °C until the RNA extraction [26]. The goat data were taken from our previous study [23].

### RNA-seq and quality control

Total RNA was obtained from hypothalamus tissue using TRIzol Reagent (Invitrogen, Carlsbad, CA, USA) following a standard extraction protocol. We monitored the contamination and degradation of RNA using 1% agarose gels. RNA was quantified using a Qubit® RNA Assay Kit in a Qubit® 2.0 Flurometer (Life Technologies, Carlsbad, CA, USA). RNA integrity was assessed using the RNA Nano 6000 Assay Kit of the Bioanalyzer 2100 system (Agilent Technologies, Santa Clara, CA, USA). For the RNA-seq analysis, 3 μg RNA was used per sample. Firstly, ribosomal RNA was removed from total RNA, and then we cleaned up the residue using ethanol precipitation. Then sequencing libraries were generated by the rRNA-depleted RNA using NEBNext® Ultra™ Directional RNA Library Prep Kit for Illumina® (NEB, USA) according to manufacturer’s recommendations. Briefly, fragmentation was carried out by using divalent cations with elevated temperature in NEB Next First Strand Synthesis Reaction Buffer (5X). Subsequently, first strand of cDNA was generated under M-MuLV Reverse Transcriptase (RNaseH-) and random hexamer primer. Second strand of cDNA was synthesised using DNA Polymerase I and RNase H. In the reaction buffer system, dUTP will replace dNTPs with dTTP. After exonuclease/polymerase treatment, remaining overhangs were changed into blunt ends. In order to prepare for hybridization, NEBNext Adaptor with hairpin loop structure were ligated under adenylation of 3′ ends of DNA fragments. The library fragments were performed with AMPure XP system (Beckman Coulter, Beverly, USA) to select cDNA fragments with 150~ 200 bp in length before PCR. Then Index (X) Primer, Universal PCR primers and Phusion High-Fidelity DNA polymerase were used for PCR. The libraries with high strand-specificity for sequencing were generated following manufacturer’s recommendations [27]. Illumina Hiseq 4000 platform was used for sequencing, as it can generate 150 bp paired-end reads. The original reads were dealt with in-house perl scripts. Low-quality reads (whose Phred scores were < 5%) were removed, yielding only clean reads. Meanwhile, we detected the quality of clean reads (Q20, Q30, and GC content) [28]. All the subsequent analyses were based on the obtained high-quality reads.

### Transcriptome assembly

We used GTF file (ftp://ftp.ncbi.nlm.nih.gov/genomes/Capra_hircus/GFF/ and ftp://ftp.ncbi.nlm.nih.gov/genomes/all/GCF/000/001/895/GCF_000001895.5_Rnor_6.0/GCF_000001895.5_Rnor_6.0_genomic.gff.gz) with annotation of the goat and rat genomes respectively. Index of the reference genome was created by Bowtie v2.0.6 [29, 30] and then we aligned paired-end clean reads to the reference genome using TopHat v2.0.9 [31]. Scripture (beta2) [32] and Cufflinks (v2.1.1) [33, 34] were adopted to assemble the reads of each sample in a reference-based approach. Both methods determined exons connectivity by spliced reads. Scripture ran using default parameters, while Cufflinks ran with min-frags-per-transfrag = 0′ and–library-type fr-firststrand’. The rest of the reference-based method concerning the reads’ assembly by default remained unchanged.

### Expression and coding potential analysis of transcripts

The FPKMs of transcripts were used to analyze the gene expression level in each sample [35]. Cuffdiff, which provides statistical routines base on the negative binomial distribution, was adopted to analyze the gene expression data [33]. Transcripts with a *P*-adjust < 0.05 were considered to have significant differential expression between two groups. Candidate lncRNAs were screened using three analytic tools, namely, CNCI (v2) [36], CPC (0.9-r2) [37], and Pfam-scan (v1.3) [38]. CNCI (v2) profiles distinguished non-coding and protein-coding sequences effectively by adjoining nucleotide triplets, the process was independent of known annotations. CPC (0.9-r2) was mainly used to detect the extent and quality of the Open Reading Frames (ORF) in a transcript, clarifying the coding and non-coding transcripts by known protein database. Each transcript was translated in all three possible frames, then any of the known protein family was identified by Pfam Scan (v1.3) in the Pfam database (release 27; adopted both Pfam A and Pfam B). The coding potential of transcripts predicted by any of the three tools above was filtered out, and those transcripts without coding potential were considered as lncRNAs for further analysis.

### Prediction of target genes and enrichment analysis

To predict the target genes of lncRNAs, protein-coding genes that were 10 K/100 K upstream and downstream of the lncRNAs were screened as potential targets [39, 40]. Pearson’s correlation coefficients with custom scripts (*r* > 0.95 or *r* < − 0.95) were used to calculate the expression levels of lncRNAs and mRNAs. Functional enrichment analysis of the lncRNA target genes from samples was performed using the DAVID platform [41]. Significance was detected by a *P*-value, calculated by the EASE score (*P* < 0.05 was considered statistically significant).

### KEGG and GO pathway analysis

Statistical enrichment was evaluated using KEGG and Gene Ontology (GO) pathway analysis. Pathway analysis of target is a functional analysis of pathways in the KEGG database (http://www.genome.jp/kegg) carried out using KOBAS software. GO enrichment of the target genes was performed by the GOseq R package and corrected by *P*-value (*P* < 0.05 were considered significantly enriched).

### Cell culture and transfection

Primary cultured hypothalamic cells were obtained from female Sprague Dawley rat (1 d). Briefly, the brains were removed and then hypothalami were dissected out from the brains. The methods of hypothalamic cell culture followed the previous research [42]. The siRNAs were designed and synthesized by GenePharma (Shanghai, China). siRNA were synthesized: sense 5’-GCAGGGACAGUCUCUGAAATT-3′, antisense 5’-UUUCAGAGACUGUCCCUGCTT-3′; negative control siRNA were synthesized: sense 5’-UUCUCCGAACGUGUCACGUTT-3′, antisense 5′- ACGUGACACGUUCGGAGAATT-3′. Lipofectamine 2000 (Invitrogen) was used in siRNAs transfection. In brief, cells were plated in 6-well plate to 50% confluence. Then 12 μl siRNA was added into 240 μl Opti-MEM medium, 6 μl of Lipofectamine 2000 into 240 μl Opti-MEM medium, and then mixed siRNA with Lipofectamine 2000. The mixture was added to each well. Cells were harvested 24 h after transfection and RNA levels detected by qRT-PCR.

### Quantitative real-time PCR

RNA-seq data was validated using qRT-PCR. We repeated qRT-PCR experiments for three times per sample from the same hypothalamic tissues. Primers were designed online using Primer 5 software and evaluated by BLAST at NCBI (Table 1). qRT-PCR was performed using SYBR green (Vazyme, China). Cycle threshold (Ct) values were used to quantify the expression levels of genes as 2^–ΔΔCT^. Expression levels were normalized to *β-actin* levels.

**Table 1:** Real-time PCR primers and sizes of the amplification products of the target and housekeeping genes

Gene	Forward primer, 5′-3′	Reverse primer, 5′-3′	Product size, (bp)
*XLOC_1041225*	GAGATGATAGCGAGATAAGAGG	GTTAGGTGACATAGTGGTTCC	113
*XLOC_1840596*	GCCTACCTAAGATTGAAGCAGTC	CAGTGAGGGAGCGAGAACC	122
*XLOC_2050950*	GCCAGCAAGAGGAAGAAGAG	CACAGAGCAGAGTTCACAGG	105
*XLOC_777127*	TGATTGTGGACCTCTAAGC	TGCGGACTCATTCTTCTG	147
*XLOC_446331*	CTCTGTTGTCTGGTCCTTC	TGCCATTGATGTCCTTACG	200
*XLOC_414038*	GGACTGACTGGCTTCTGAG	TGGCTGTGCTGGATTGAC	140
*XLOC_181783*	TGCTGATTCTCCCTGTGGTTTATG	GCTTCCCTGGTTGTGCTTGG	104
*XLOC_113511*	CTTTCTCCTCCCGCTCTG	TCTGATCTCGCTGTCTTCG	132
*Igfbp5* (goat)	CCCAACTGTGACCGCAAAG	TCCACGCACCAGCAGATG	86
*Igfbp6* (goat)	TACAAGACACTGAGATGG	CTATGGTCACAATTAGGC	115
*Tgfbi* (goat)	GTGCCCGCCTGCTGAAAG	TGCTGGATGTTGTTGGTGACG	94
*Kiss-1* (goat)	GCCGCTGTTGTTCTGTTGAC	CTGGGGTTCTGCCATTTGA	117
*Igfbp5* (rat)	CAGTCGTGTGGCGTCTAC	AGCGGCTTCTCCTCATCC	77
*Igfbp6* (rat)	CCGCAGACACTTGGATTCAG	TCACAGTTTGGCACATAGAGC	87
*Tgfbi* (rat)	CAGTCGTGTGGCGTCTAC	AGCGGCTTCTCCTCATCC	163
*Kiss-1* (rat)	TGCTGCTTCTCCTCTGTG	CCAGGCATTAACGAGTTCC	116
*Actb* (goat)	CGTGACATCAAGGAGAAG	GAAGGAAGGCTGGAAGAG	171
*β-actin* (rat)	CCCATCTATGAGGGTTACGC	TTTAATTGTCACGCACGATTTC	150

### Statistical analysis

Further analysis of RNA-seq data was performed using the statistical R package (ggplot2, DESeq, edgeR, and DEGSeq; R, Auckland, NZL), as well graphical representations, adopting multiple testing. SPSS 17.0 software package (SPSS, Chicago, IL, USA) was applied to analyze the qRT-PCR data. Significance of data was defined at *P* < 0.05.

## Results

### Comparison of the features of goat and rat lncRNAs

**Fig. 1:**
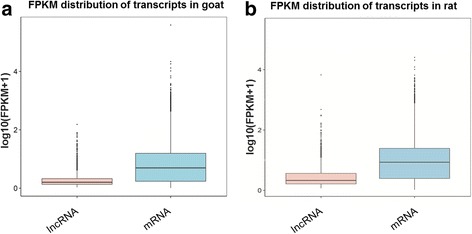
Comparison of transcript expression levels between goats and rats. Expression level of mRNAs and lncRNAs were indicated by log_10_(FPKM + 1). **a**. FPKM distribution of transcripts in goat, **b**. FPKM distribution of transcripts in rat

**Fig. 2:**
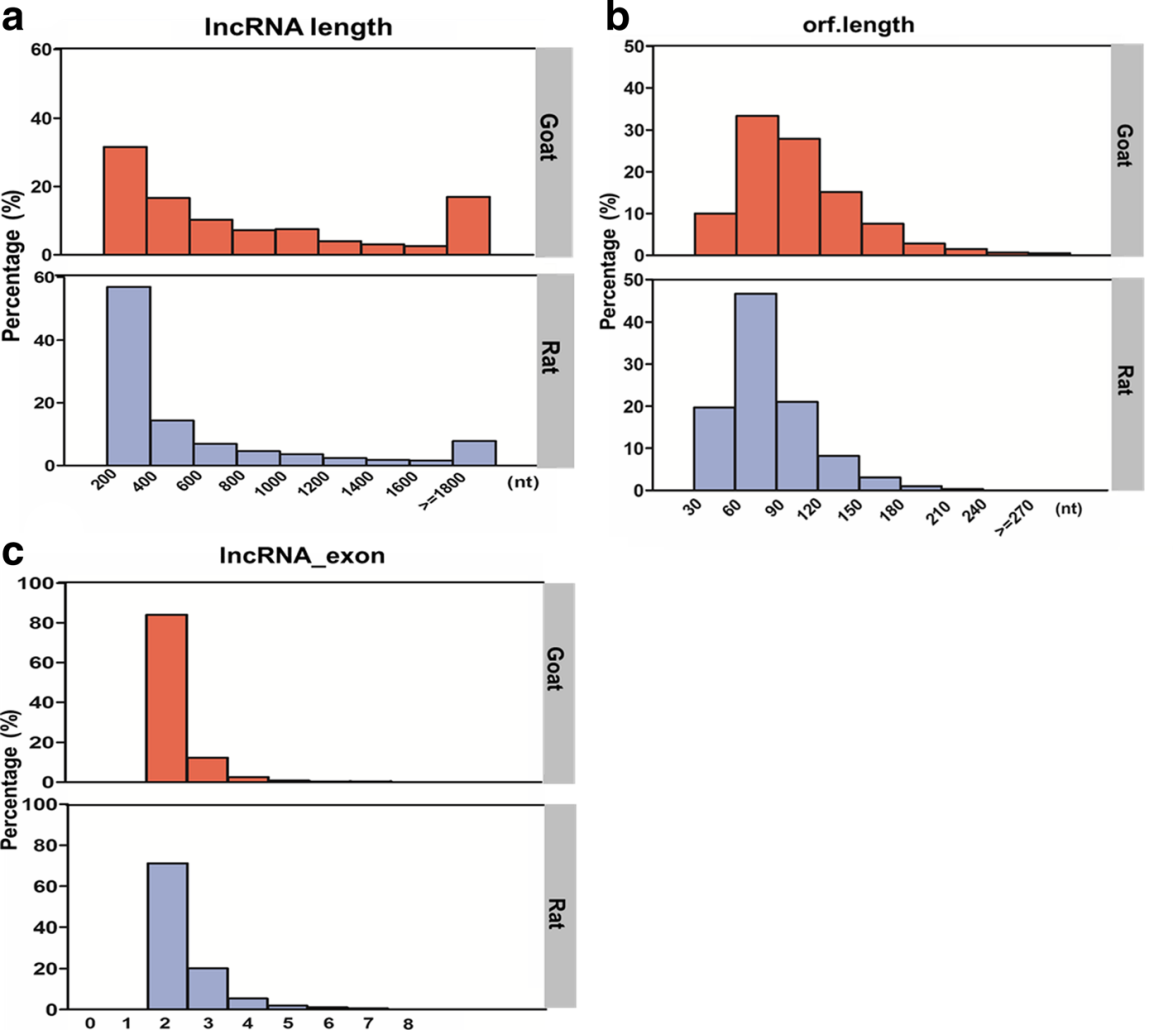
Comparison of features of lncRNAs in goats and rats. **a** Length distribution of lncRNAs between the two species. **b** Distribution of open reading frame length between the two species. **c** Distribution of exon number of lncRNAs between the two species

RNA-seq was performed to investigate the expression of lncRNAs in puberty. By analysis between rats and the previous goats RNA-seq data, the lncRNAs expression was lower (*P* < 0.05) than mRNAs expression in both goat and rat hypothalamus (Fig. 1). The lncRNAs length in the hypothalamus of goats was approximately 200–400 nt, and accounted for 31.6% of the total lncRNAs; In rat, the length of lncRNAs in ranged from 200 to 400 nt, and accounted for 56.9% of lncRNAs, it was greater (*P* < 0.05) than those in goat (Fig. 2a). The lncRNAs whose ORF length ranged from 61 to 90 nt accounted for 33.4% of the total lncRNAs in goat; In rat, lncRNAs whose ORF length ranged from 61 to 90 nt accounted for 46.7% of the total lncRNAs, it was higher (*P* < 0.05) than those in goat (Fig. 2b). Moreover, most lncRNAs contained two exons in pubertal goat and rat, and accounted for 84% and 71.2% in both animals, respectively. The proportion of lncRNAs contained two exons was greater (*P* < 0.05) in goat than those in rat (Fig. 2c).

### Analysis of target genes between goats and rats

We used the cis model to screen protein-coding genes that were 10 K/100 K upstream and downstream of the lncRNAs as potential lncRNA targets.

**Fig. 3:**
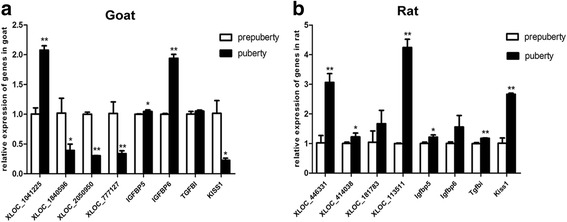
Results of qRT-PCR of the candidate genes involved in puberty. The lncRNAs and target genes were examined using quantitative qRT-PCR. Data are expressed as the means ± SD (*n* = 3). *P < 0.05, **P < 0.01. **a**. Goat, **b**. Rat

Among the differentially expressed target genes (Additional file 2), four were common to both pubertal goat and rat. These genes were the potential targets of *XLOC_1041225*, *XLOC_1840596*, *XLOC_2050950*, and *XLOC_777127* in goat and *XLOC_446331*, *XLOC_414038*, *XLOC_181783*, and *XLOC_113511* in rat. Gene expression levels were detected by quantitative PCR (Fig. 3). The results revealed that the expression levels of *XLOC_1041225* and its target gene *Igfbp5* in goat and *XLOC_446331* and its target gene *Igfbp5* in rat significantly increased in puberty (*P* < 0.05). We hypothesized that *Igfbp5* may be a key regulator in female puberty. Moreover, *XLOC_777127* and its target *Kiss-1* in goat, and *XLOC_113511* and its targets *Kiss-1* in rat also showed significant differential expression. However, the *lncRNA* and *Kiss-1* expression profile significantly differed between the two species (*P* < 0.05). Among the remaining genes screened in this study, there were no similarities in expression profiles between goat and rat as the results of quantitative PCR showed.

### KEGG and GO analysis of predicted target genes

KEGG pathway analysis of lncRNA targets revealed that a total of 90 and 152 terms were enriched in goat and rat, respectively. Moreover, of these, 66 terms were enriched in both species (Additional file 3). The common terms included the AMPK signaling pathway, oxytocin signaling pathway, and insulin secretion, which are related to puberty and reproduction. Some terms were specifically enriched in either species (*P* < 0.05). For instance, the glycosaminoglycan degradation signaling pathway was significantly enriched in pubertal goats alone and ribosome signaling pathway was significantly enriched in pubertal rats alone.

GO analysis of the predicted target genes showed 73 terms and 528 terms that had been significantly enriched in goat and rat, respectively (*P* < 0.05). Three common enriched terms were discovered (Additional file 4), of which the enriched GO term pheromone receptor activity was involved with estrus of goat.

### KEGG and GO analysis of mRNAs

**Fig. 4:**
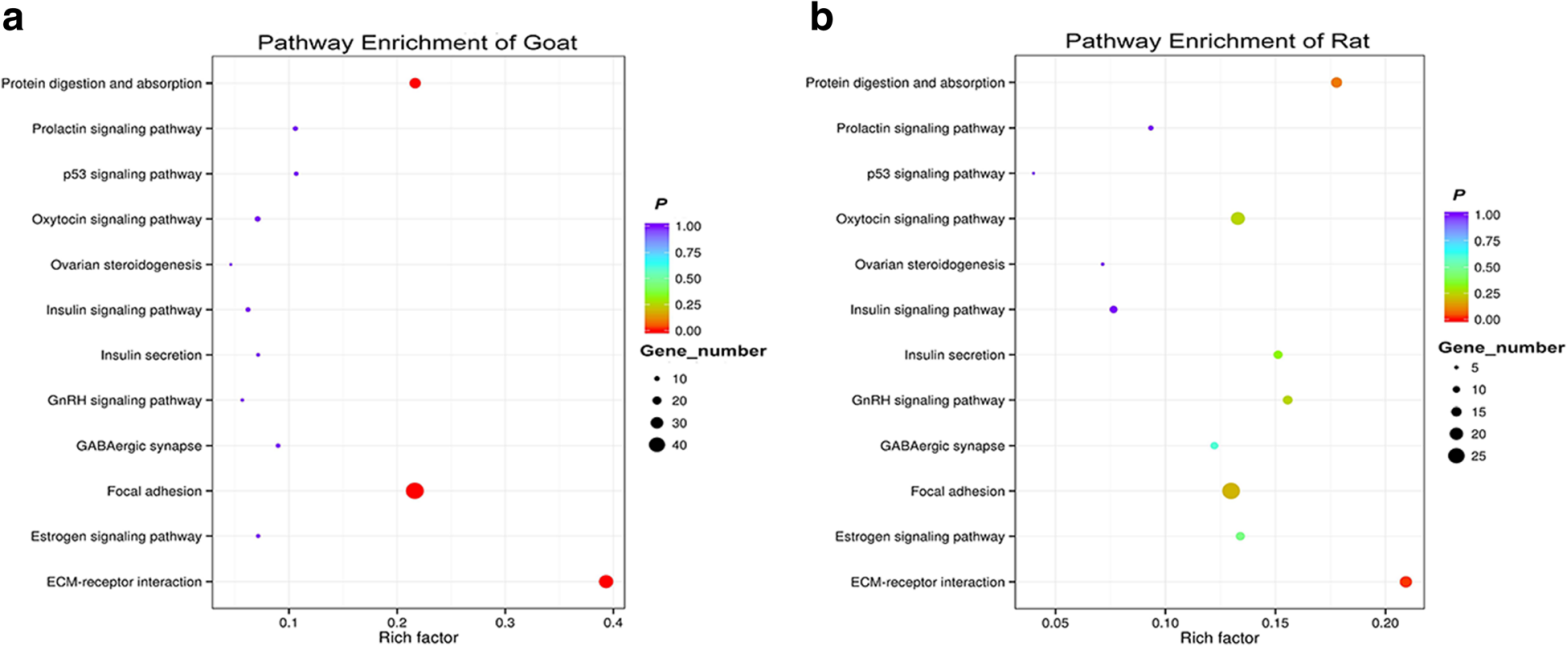
Analysis of KEGG pathways for mRNA between goats and rats. Y-axis, the terms of pathways; X-axis, rich factor; the size of point indicated the number of differential expressed genes. The colour of point indicated the range of *P*. **a** KEGG pathways between prepubertal and pubertal goats. **b** KEGG pathways between prepubertal and pubertal rats

**Fig. 5:**
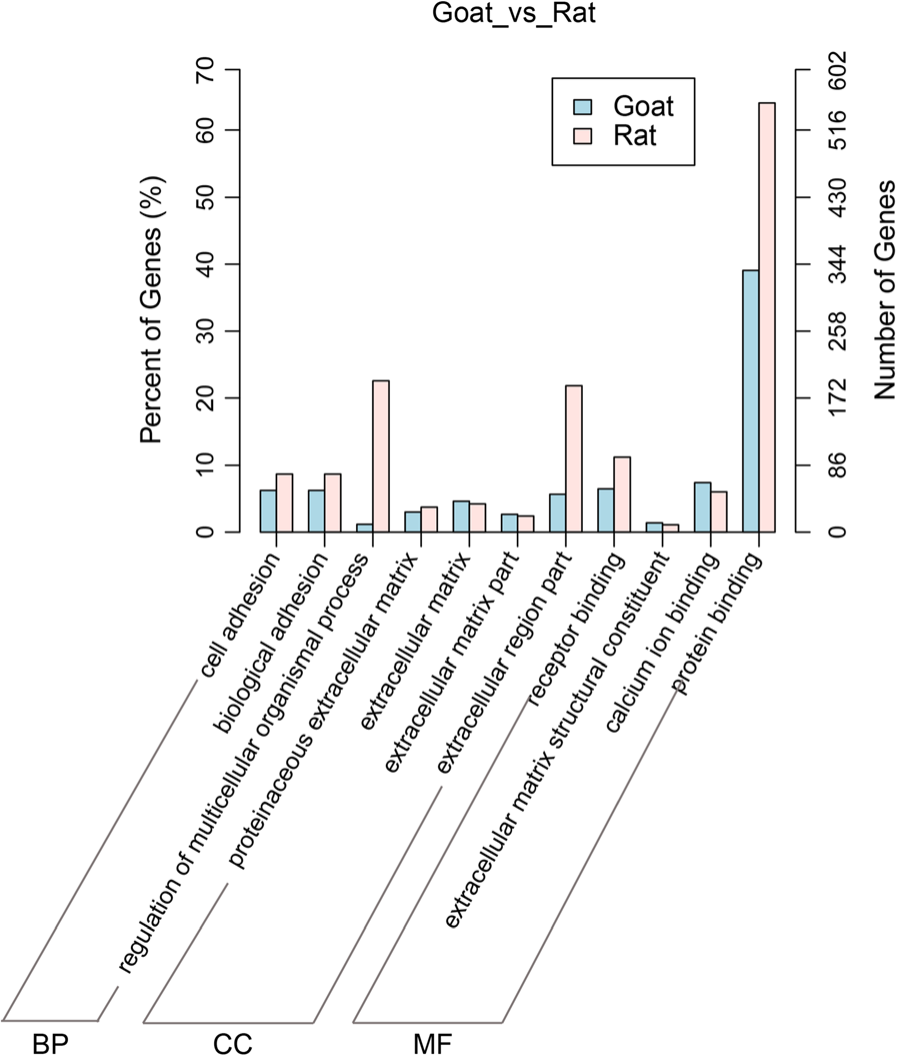
Analysis of GO pathways involved in puberty between goat and rat. BP, biological process; CC, cellular component; MF, molecular function. Left Y-axis, the ratio of single annotation in total annotation. Right Y-axis, The number of genes included in single annotation. X-axis, the terms of GO pathways

In order to obtain further update of regulation of puberty, KEGG pathway analysis was also performed between protein-coding genes in goat and rat. We analyzed 12 common enriched terms (Fig. 4, Additional file 5). ECM-receptor interaction signaling pathway was significantly enriched (*P* < 0.05). Moreover, numerous signaling pathways related to puberty and reproduction were enriched, such as estrogen signaling pathway, GABAergic synapse, GnRH signaling pathway, insulin secretion, ovarian steroidogenesis, and so on (Fig. 4). Otherwise, the GO analysis results of the protein-coding genes in goat and rat show 11 common significantly (*P* < 0.05) enriched terms (Fig. 5, Additional file 6). These enriched signaling pathways may be involved in puberty and reproduction.

### The verification of candidate lncRNA-*XLOC_446331*

**Fig. 6:**
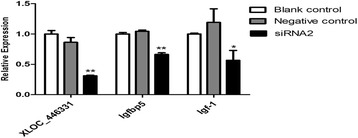
The expression of genes in hypothalamus cells after siRNA transfection. The expressions of genes were examined using quantitative qRT-PCR. Data are expressed as the means ± SD (*n* = 3). *P < 0.05, **P < 0.01

In order to investigate the roles of candidate lncRNA-*XLOC_446331*, siRNA transfection experiment was performed in hypothalamic cells. Results indicated that the expressions of lncRNA-*XLOC_446331* was remarkably inhibited (*P* < 0.01) by siRNA compared to the Negative Control (Fig. 6). The levels of *Igfbp5*, as the target gene of lncRNA-*XLOC_446331*, was also significantly decreased (*P* < 0.01). Furthermore, we measured the expression of downstream gene *Igf-1* which was involved in femal puberty. The expression of *Igf-1* was also significantly suppressed (*P* < 0.05) after silencing *XLOC_446331* by siRNA (Fig. 6).

## Discussion

We performed the RNA-seq analysis between goat and rat to investigate the features of lncRNAs in puberty between these two species. In hypothalamus, overall expression levels of lncRNAs was lower than mRNA in both goats and rats, and the results were in accordance with previous research [43]. The length of the lncRNAs, the length of the ORF, and the exon count were also compared between the two species. Similar to previous lncRNA studies in chicken and pig [43, 44], our study revealed that most of the lncRNAs in rats were in the range of 200 ~ 400 nt but a lower percentage of lncRNAs in goats were in this size range (*P* < 0.05). Moreover, the length of ORF and exon count in lncRNAs from rat were both shorter and lower than the corresponding values in goat (*P* < 0.05). The results indicated that the features of lncRNAs in the hypothalamus of these two species in puberty differed slightly, and total distribution was consistent between the two species in features of lncRNAs. The latter may indicates that conserved functions of differentially expressed lncRNA between the rats and goats, exist.

Over the last few years, a number of studies have revealed that lncRNAs function on neighboring protein-coding genes. *GnRH-E1 RNA*, a novel lncRNA transcribed from *Gnrh1* enhancer, was found to play an important role in the maturation of GnRH neurons [45]. The *lncRNA Neat1*, which was highly expressed in luteal tissue, was essential for the establishment of pregnancy by regulating corpus luteum formation in mice [46, 47]. Therefore, roles of lncRNAs in mammals can be predicted by identifying the roles of neighboring protein-coding genes. In order to investigate the lncRNAs that were involved in puberty, four common lncRNAs target genes were discovered by simultaneous RNA-seq analysis of goat and rat hypothalamus. Among the common target genes *Igfbp5*, *Igfbp6*, *Tgfbi*, and *Kiss-1* were involved in mammalian reproduction and puberty.

As previously reported, many studies have revealed that insulin-like growth factors (IGFs) play important roles in reproduction and puberty. For example, as IGF-1 can affect hypothalamic GnRH release, the time of puberty can be significantly advanced by IGF-1 stimulation in female rats [48]. The IGF-1 pathway also regulated the age of puberty in Brahman cattle [49]. Moreover, IGFBP5 has been shown to play important roles in the development of the anterior pituitary gland in both male and female rats [50, 51]. In the present study, lncRNA *XLOC_1041225* (in goat), *XLOC_446331* (in rat) and their common target gene *Igfbp5* were significantly upregulated in puberty which indicated the network lncRNA-*IGFBP5* may be crucial regulator in onset of female puberty.

Several studies have confirmed the association of Kiss-1/GPR54, which stimulates GnRH secretion with puberty onset [25, 52, 53]. The results of the quantitative PCR showed an increase in the lncRNA *XLOC_113511* and its neighboring target gene *Kiss-1* in rat. However, the lncRNA *XLOC_777127* and its target *Kiss-1* were decreased in goat. We hypothesized that this difference in expression may be due to the different sampling times and species.

Among the 66 common terms, several KEGG pathways participated in the onset of puberty, including the AMPK signaling pathway, oxytocin signaling pathway, and insulin secretion. As showed previously, the AMPK signaling pathway was a novel regulator of GnRH release, which played an important role in regulating puberty and reproduction [54, 55]. Otherwise, GnRH release was accelerated by oxytocin acting on the prepubertal female hypothalamus, and the onset of female puberty was advanced [56]. Moreover, studies have reported that insulin plays a role in the control of the HPG [57]. Lack of insulin receptors in Kiss-1 neurons has been shown to delay puberty in female and male mice [8]. Thus, the lncRNAs which were screened in the present study may perform a crucial role in regulation of puberty via the above KEGG pathways. Moreover, the pheromone receptor activity signaling pathway, which relates to goat estrus [58], was significantly enriched in our GO analysis between goat and rat.

In order to obtain more information about regulation of puberty, KEGG and GO enrichment analysis was performed on differential expressed mRNAs in goat and rat. The results of KEGG analysis revealed several key signaling pathways, such as estrogen signaling pathway, GABAergic synapse, GnRH signaling pathway, insulin secretion, and ovarian steroidogenesis signaling pathway. These above KEGG pathways have been suggested to participate in the regulation of female puberty [59]. Otherwise, 11 common significantly enriched terms, as shown in the GO analysis of differential expressed mRNAs, may be indirectly involved in regulation of puberty.

As previous research, the regulator of IGF-1 play important roles in regulating puberty and nutritional control of reproduction [21, 60]. Furthermore, IGFBP5 was the binding protein of IGF-1, and which performed a crucial role in development of the anterior pituitary gland. Therefore, we performed siRNA transfection experiment to verify the roles of candidate lncRNA-*XLOC_446331* and *Igfbp5*. The expression of *Igfbp5* and the downstream genes *Igf-1* was significantly decreased by the interference of siRNA target to *XLOC_446331*. As previous research shown, the development of endometrium was controled by lncRNA-H19 via IGF signaling [61]. Therefore, we inferred the lncRNA *XLOC_446331* may participate in regulating female puberty by IGF-1 pathway. However, further research is needed in this regard.

## Conclusion

In conclusion, the present study analysed the role of lncRNAs from hypothalamus in regulation of female puberty. Using RNA-seq analysis, we discovered the similar genomic features and expression profile of lncRNAs in puberty between rat and goat. Furthermore, several biological processes associated with female puberty were annotated by KEGG and GO analysis of lncRNAs target genes and differentially expressed mRNA. Moreover, our results showed the candidate lncRNA *XLOC_446331* may play a crucial role in regulating female puberty. These results indicated that female reproduction and puberty were regulated by complex networks of lncRNAs and mRNAs, and our research provides a resource for lncRNA studies in puberty as well as the regulation in female estrus cycle.

## Additional files

Additional file 1: The identification of the first estrous cycle phase. (DOCX 4296 kb)

Additional file 2: Prediction of target genes of lncRNAs. (XLSX 88 kb)

Additional file 3: KEGG terms of predicted target genes. (XLSX 28 kb)

Additional file 4: GO terms of predicted target genes. (XLSX 11 kb)

Additional file 5: KEGG terms of mRNAs. (XLSX 18 kb)

Additional file 6: GO terms of mRNAs. (XLSX 23 kb)

## Abbreviations

GnRH: gonadotropin-releasing hormone
GO: Gene Ontology
HPG: hypothalamic-pituitary-gonadal
Igf-1: insulin-like growth factor 1
Igfbp5: insulin-like growth factor binding protein 5
KEGG: Kyoto Encyclopedia of Genes and Genomes
LncRNA: long noncoding RNA
