# Supplementary material for: RNA-seq analysis of lncRNA-controlled developmental gene expression during puberty in goat & rat
Source: BMC Genet. 2018 Apr 2;19:19. doi: 10.1186/s12863-018-0608-9 (PMC5879571; doi:10.1186/s12863-018-0608-9)
Supplement: Supplementary file 1 — The identification of the first estrous cycle phase. (DOCX 4296 kb) [file 12863_2018_608_MOESM1_ESM.docx]

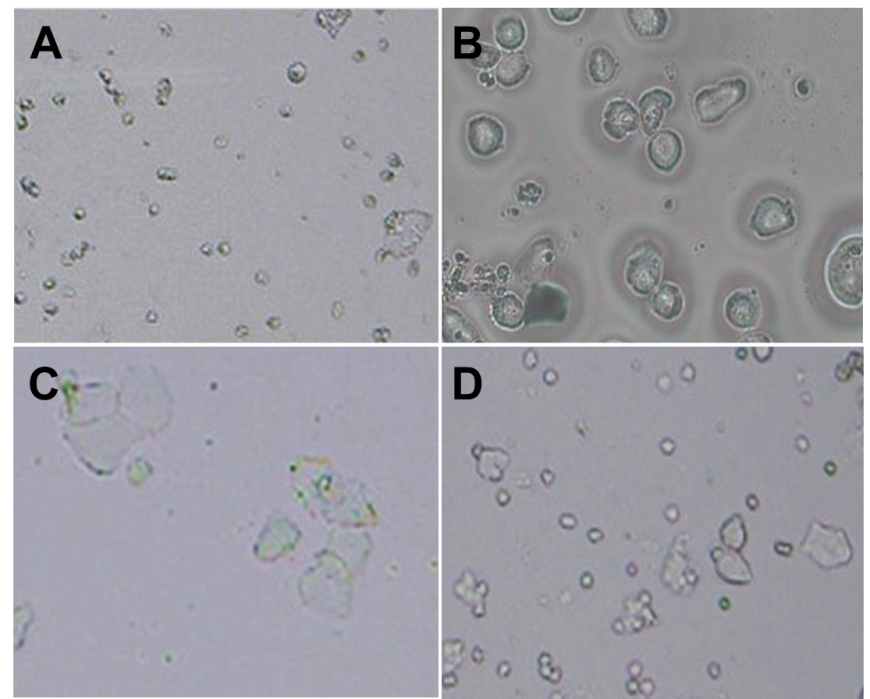


Fig. 1 The estrous cycle phase of female rats were determined by the cellular types from the vaginal smear. Photomicrographs of vaginal smears from female rats during the first estrous cycle. (A) diestrus phase (a predominance of leukocytes); (B) proestrus (a predominance of nucleated epithelial cells), (C) estrus (a predominance of cornified cells), and (D) metaestrus (the same proportion among leukocytes, cornified, and nucleated epithelial cells). The rats in first estrus was defined as pubertal material.


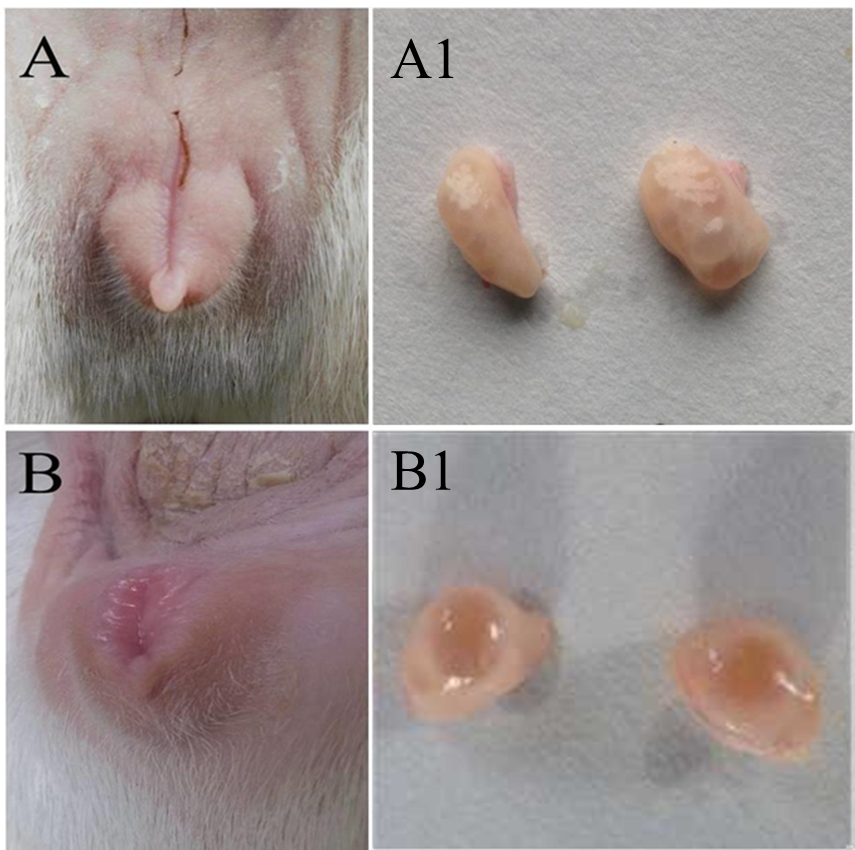


Fig. 2 The vulva of female goats appear swollen, red, and secreting mucus in the first estrus (B), compared with prepuberty (A). In addition, healthy dominant follicles were existed in the first estrus stages ovary (B1) , that were larger than those of follicles in prepuberty (A1).
